# A small molecule screen identifies *in vivo* modulators of peripheral nerve regeneration in zebrafish

**DOI:** 10.1371/journal.pone.0178854

**Published:** 2017-06-02

**Authors:** Juliane Bremer, Julianne Skinner, Michael Granato

**Affiliations:** Department of Cell and Developmental Biology, Perelman School of Medicine, University of Pennsylvania, Philadelphia, Pennsylvania, United States of America; Deakin School of Medicine, AUSTRALIA

## Abstract

Adult vertebrates have retained the ability to regenerate peripheral nerves after injury, although regeneration is frequently incomplete, often leading to functional impairments. Small molecule screens using whole organisms have high potential to identify biologically relevant targets, yet currently available assays for *in vivo* peripheral nerve regeneration are either very laborious and/or require complex technology. Here we take advantage of the optical transparency of larval zebrafish to develop a simple and fast pectoral fin removal assay that measures peripheral nerve regeneration *in vivo*. Twenty-four hours after fin amputation we observe robust and stereotyped nerve regrowth at the fin base. Similar to laser mediated nerve transection, nerve regrowth after fin amputation requires Schwann cells and FGF signaling, confirming that the fin amputation assay identifies pathways relevant for peripheral nerve regeneration. From a library of small molecules with known targets, we identified 21 compounds that impair peripheral nerve regeneration. Several of these compounds target known regulators of nerve regeneration, further validating the fin removal assay. Twelve of the identified compounds affect targets not previously known to control peripheral nerve regeneration. Using a laser-mediated nerve transection assay we tested ten of those compounds and confirmed six of these compounds to impair peripheral nerve regeneration: an EGFR inhibitor, a glucocorticoid, prostaglandin D2, a retinoic acid agonist, an inhibitor of calcium channels and a topoisomerase I inhibitor. Thus, we established a technically simple assay to rapidly identify valuable entry points into pathways critical for vertebrate peripheral nerve regeneration.

## Introduction

Insults such as physical trauma, chemotherapy or metabolic disorders can lead to peripheral nerve damage [1, 2]. Given the diversity of insults, it is not surprising that peripheral nerve damage has a high incidence rate that is even higher in the elderly population [3–5]. Unlike the central nervous system (CNS), the peripheral nervous system has retained considerable capacity for axonal regeneration. This is in part due to the ability of PNS neurons to mount a cell-intrinsic growth-promoting response and to an environment amiable to axonal regeneration [6]. Yet despite their remarkable ability for regeneration, functional regeneration often remains incomplete [1, 7]. Therefore, novel treatment strategies to enhance the robustness and fidelity of peripheral nerve regeneration would be beneficial.

Small molecule screens in zebrafish have been highly successful to identify bioactive compounds that control various biological processes including rest/wake regulation [8], cancer [9, 10], hair cell regeneration [11], metabolism [12], haematopoietic stem cell homeostasis [13], and BMP signaling [14]. We have recently developed a laser mediated nerve transection assay to visualize and quantify peripheral nerve regeneration in larval zebrafish [15, 16]. This assay has yielded invaluable insights into the cellular and molecular mechanisms that promote axon regrowth and directionality [15–18]. While powerful, this assay is limited as a screening tool by its very low throughput. To circumvent this limitation, we developed a simple and fast assay to monitor regeneration of peripheral nerves innervating pectoral fins. After pectoral fin removal, we assessed axonal regrowth at the fin base. Using this high throughput *in vivo* assay we screened over 340 compounds with known targets which led to the identification of several targets previously not implicated in axonal regeneration.

## Results

### Pectoral fin removal induces robust nerve regeneration

**Fig 1:**
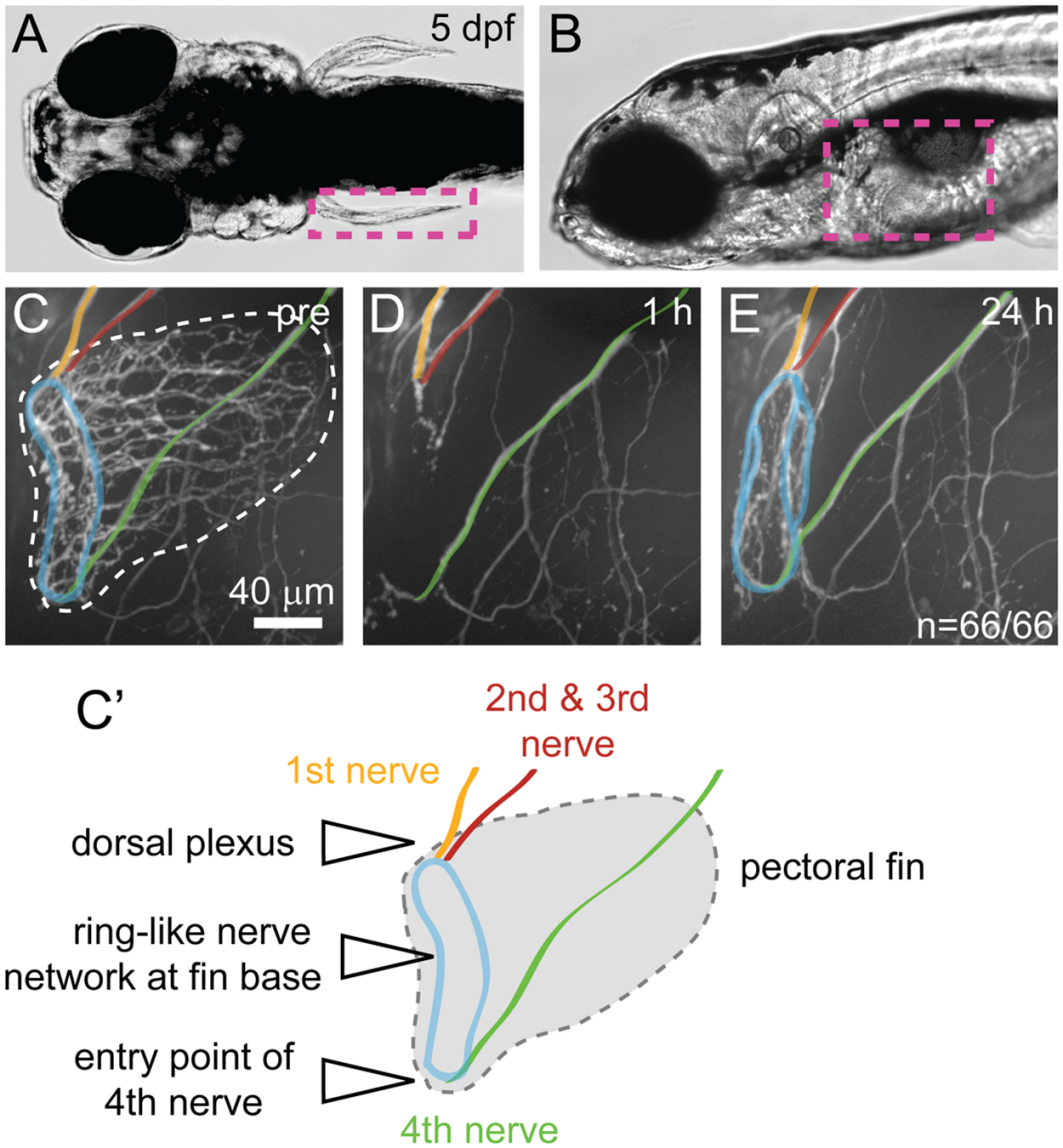
Pectoral fin innervation and nerve regrowth after fin removal. (A-B) Dorsal (A) and side view (B) of a larval zebrafish at 5 dpf showing the location of the pectoral fin (magenta dashed box). (C, C') Pectoral fin innervation in a *Tg(mnx1*:*GFP)* transgenic larva at 5 dpf (C) and a schematic (C'). The first segmental nerve (orange) forms the dorsal plexus together with the joint nerves 2 and 3 (red). The fourth nerve (green) travels along the larval body wall, before it enters the pectoral fin ventrally. These nerves contribute to the ring-like network at the fin base (blue). (D-E) Fin region of *Tg(mnx1*:*GFP)* transgenic larvae 1 hour (D) and 24 hours (E) after fin removal. One hour after the pectoral fin has been removed using tweezers, GFP positive axons at the fin base are no longer detectable, leaving behind the stumps of nerve 1–3 (orange, red) at the dorsal plexus and at the 4th nerve entry point (green, D). Twenty-four hours after fin removal, GFP positive axons at the fin base have regrown robustly, reforming the ring-like nerve network at the fin base (blue) in 100% of the 66 control fish tested (untreated and DMSO treated, E).

In zebrafish larvae the pectoral fins are innervated by the four anterior-most segmental spinal nerves, comprised predominantly of motor axons. The first three segmental nerves form a plexus at the dorsal-most aspect of the fin and the fourth nerve travels along the body wall before it enters the fin at the ventral-most aspect [19, 20]. All four nerves contribute to a ring-like nerve network at the fin base from which several branches extend to innervate the fin musculature (Fig 1A–1C) [19, 20]. Using tweezers, we severed the pectoral fin including the ring-like nerve network at the fin base (Fig 1D). Importantly, this surgery leaves behind a proximal nerve stump located at the dorsal plexus and the ventral-most aspect of the fin (Fig 1D). By 24 hours post fin removal, regenerating *Tg(mnx1*:*GFP)* positive axons had re-established the ring-like nerve network at the fin base (Fig 1E). Regeneration was remarkably robust resulting in the formation of a ring-like nerve network in 100% of pectoral fins (n = 66/66, Fig 1E). Moreover, surgery and regeneration analysis including post-injury imaging was rapid, allowing analysis of about 160 pectoral fins within 2 days. Thus, following pectoral fin removal we observed robust axonal regrowth *in vivo*.

### Amputation induced nerve transection exhibits key features of vertebrate peripheral nerve regeneration

**Fig 2:**
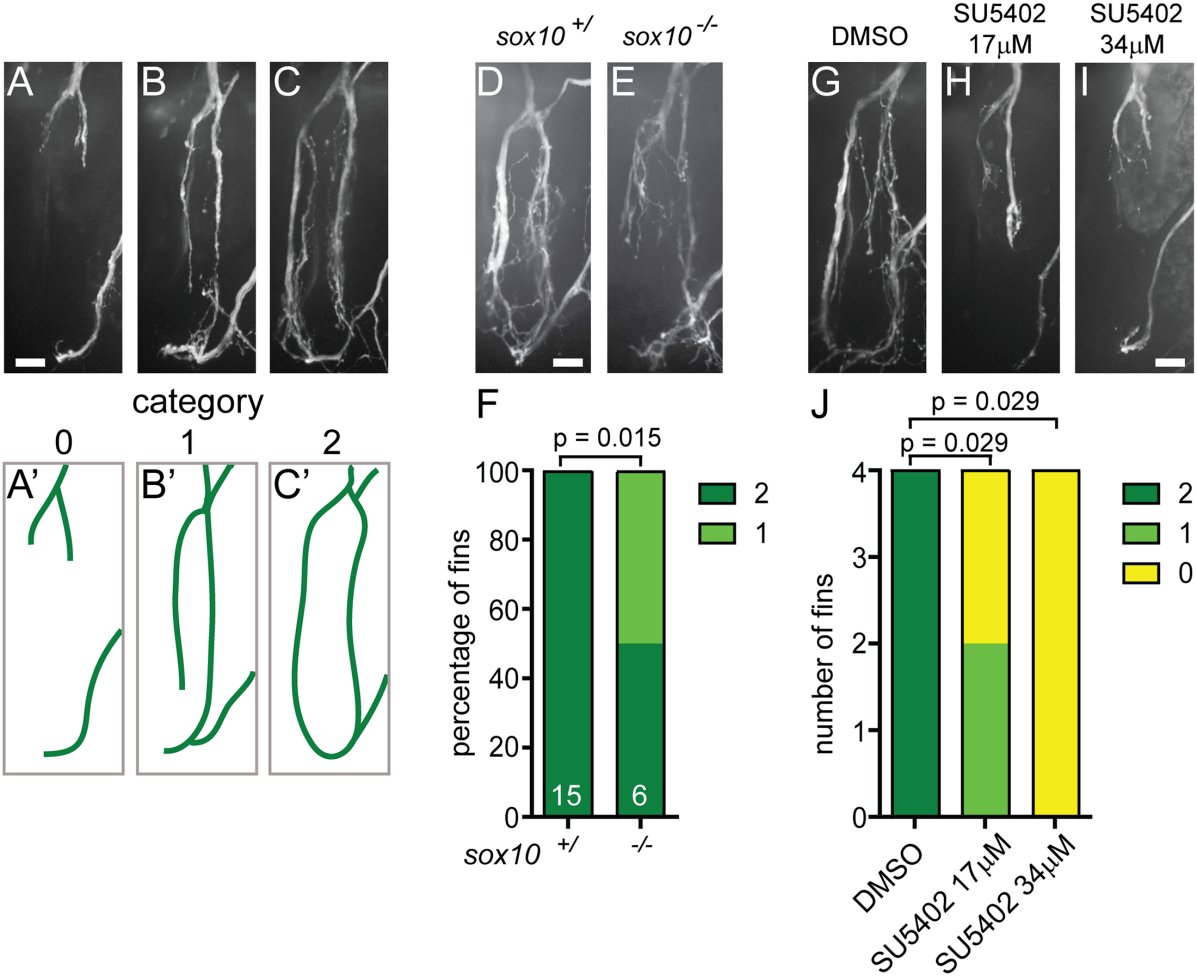
Nerve regrowth at the fin base requires *sox10* and FGF. (A-C) Quantification of fin nerve regrowth in *Tg(mnx1*:*GFP)* transgenic larvae 24 hours after fin removal; nerves show different degrees of nerve regrowth (A-C). Categories 0, 1, and 2 indicate that none (A, A'), one (B, B'), or both sides (C,C') of the ring-like nerve network at the fin base regrew, respectively. (A'-C'): schematic representation of these results. (D-F) Nerve regrowth at the fin base 24 hours after fin removal in a wild type sibling (D) and a *sox10* mutant (E). *Sox10* mutants display significantly reduced nerve regrowth compared to wild type siblings. Graphical representation of the extent of nerve regrowth (category 0, 1, 2; F). (G-J) Nerve regrowth at the fin base at 24 hours after fin removal in a control larva treated with DMSO (G) and larvae treated with a low (17μM, H) or a high dose (34μM, I) of the FGF inhibitor SU5402. DMSO or SU5402 were added immediately after fin amputation. FGF inhibitor-treated larvae show significantly less nerve regrowth compared to DMSO-treated controls. Graphical representation of the extent of nerve regrowth (category 0, 1, 2; J). All scale bars are 10 μm.

Fin removal leaves behind an open wound and constitutes an amputation injury. This is in contrast to nerve crush or nerve transection injuries, and therefore prompted us to determine if and to what extent the cellular and molecular aspects of nerve regrowth after fin removal resembles those of transection-induced nerve regeneration. We therefore tested whether Schwann cells and FGF signaling play as important roles in peripheral nerve regeneration after fin amputation as observed following crush injury in rodents or laser mediated nerve transection in zebrafish [16, 18, 21–25]. To quantify the extent of axon regeneration at 24 hours after fin removal, we applied a three-category rubrics illustrated in Fig 2A–2C. We have previously shown that genetic ablation of Schwann cells in *sox10* mutants or acute ablation of Schwann cells severely compromises spinal motor nerves following nerve transection [16]. In contrast to wild type siblings, regenerating axons in fin amputated *sox10* mutants failed to reform the ring-like nerve network at the fin base in 50% of the fins examined and stalled prematurely (Fig 2D–2F). Similar to the dependence on Schwann cells, FGF signaling has been shown to promote peripheral nerve regeneration in rodents [21–25]. We observed significantly and dose-dependently reduced axon regrowth at the fin base in larvae treated with the FGF inhibitor SU5402 (Fig 2G–2J). Thus, axonal regeneration following fin amputation requires the presence of Schwann cell as well as FGF signaling, two hallmarks of peripheral nerve regeneration.

### A small molecule screen for compounds that control amputation induced nerve regeneration

**Fig 3:**
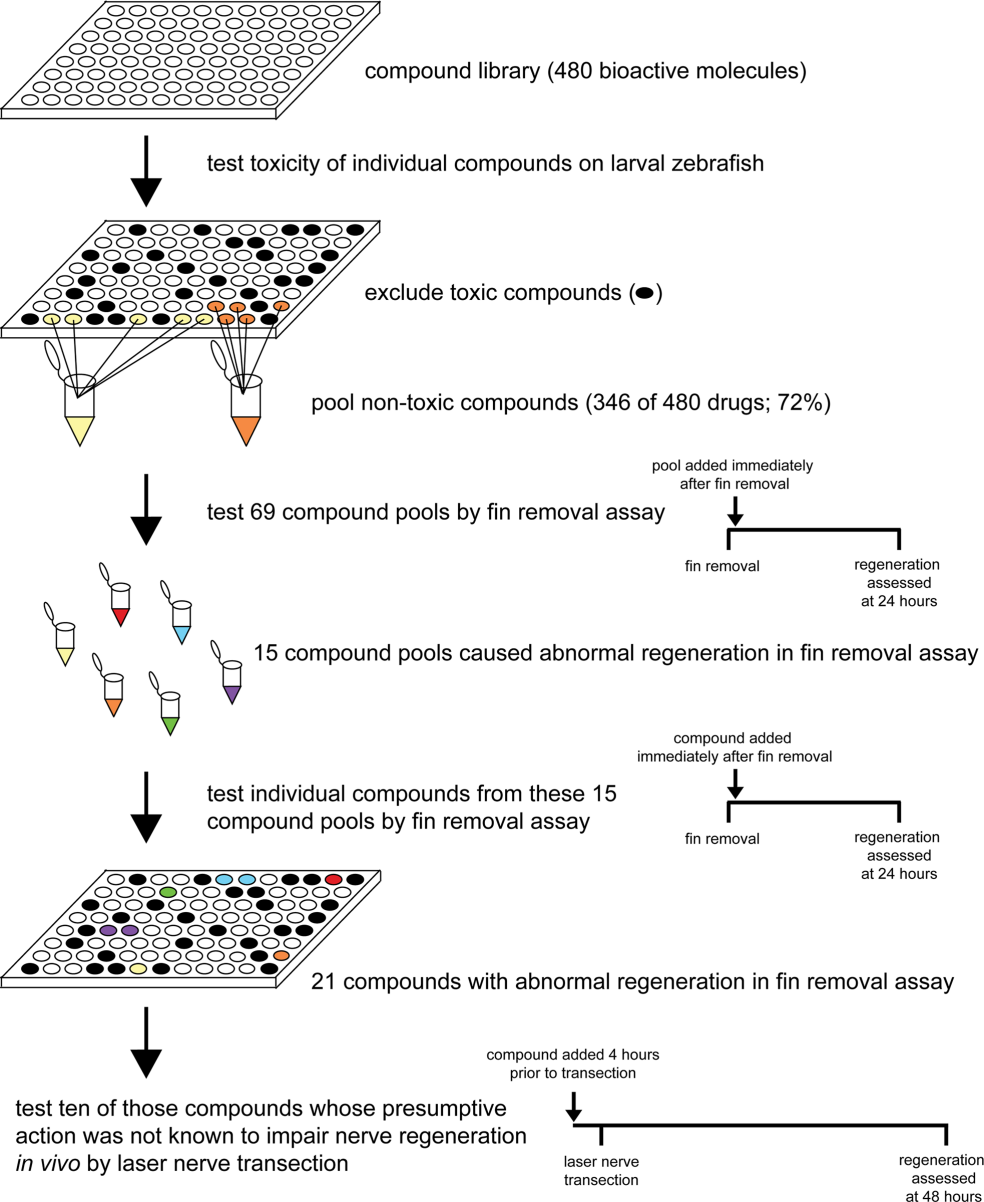
Small molecule screen workflow. For simplicity a single 96-well plate containing active compounds is shown. First we tested the toxicity of the individual compounds on larval zebrafish and excluded 134 toxic compounds from further analysis (black wells). We chose to apply the compounds at a uniform dilution of 1:1,000 since this ensured a fast throughput and a relatively low percentage of toxic compounds. Depending on the stock concentration, most compounds were tested at a final concentration of 1 μM or 5 μg/ml with some exceptions (S1 Table). 346 remaining compounds were assigned to 69 pools. Here, examples of two pools are shown (yellow and orange). In a first pass, we tested the 69 compound pools for their effect on nerve regrowth in the fin removal assay and found 15 pools that affected nerve regrowth (differently colored pools). Then we tested the individual compounds from these 15 pools in a second pass and identified 21 individual compounds that affected nerve regrowth in the fin removal assay. Ten of these compounds were further tested using laser nerve transection. Timeline diagrams of treatment after fin removal and laser nerve transection are shown on the right side.

Having established a robust and rapid assay with hallmarks of vertebrate peripheral nerve regeneration, we next screened the ICCB Known Bioactives library (Enzo), containing 480 bioactive compounds with known biological targets to identify molecular pathways promoting nerve regrowth. We first screened the library for effects on survival and excluded 134 compounds that compared to 1% DMSO exposure diminished overall health (see Material and methods for details) over the course of 48 hours. We then combined the remaining 346 compounds into 69 distinct pools (Fig 3, S1 Table). Each of these pools was then added to a group of four larvae immediately following fin amputation. In DMSO treated controls, regenerating axons re-formed a ring-like nerve network at the fin base 24 hours after fin removal (Figs 1E and 2G). In larvae exposed to fifteen pools, regenerating axons failed to form the characteristic ring-like network. Specifically, seven pools predominantly caused defasciculated regrowth while eight pools predominantly reduced regrowth (Fig 3, S1 Table). We next tested each of the compounds within a given pool individually. In three of the pools we failed to identify a singly effective compound. In the remaining 12 pools we identified 1–3 compounds per pool totaling 21 compounds which caused impaired axonal regrowth (Fig 3, Table 1, S1 Table). Seven compounds inhibited pathways such as the MAPK pathway previously shown to promote axon regeneration *in vivo*, further demonstrating the validity of the fin removal assay (Table 1, S1 Table). Of the remaining 14 compounds, four impaired regeneration while the presumptive action of the compound had previously been reported to promote regeneration. These compounds included the retinoic acid (RA) agonists AM-580 and 9-cis-retinoic acid, the corticosteroid signaling agonist dexamethasone, and the calcium channel blocker verapamil (Table 1). Finally, we identified ten compounds whose presumptive action was previously not known to control peripheral nerve regeneration *in vivo*. Two compounds (C2 dihydroceramide and tyrphostin-1) have no known targets and were not further tested. The remaining eight compounds included the topoisomerase I inhibitor 10-Hydroxycamptothecin (10-HCT), the fatty acid 9(S)-HpODE, the cannabinoid receptor agonist anandamide (22:4,n-6), the cyclic nucleotide-dependent protein kinase inhibitor HA-1004, the EGFR inhibitor lavendustin A, the SOD mimetic MnTBAP, the TRPP3 channel inhibitor phenamil, and prostaglandin D2 (PGD2) (Table 1).

**Table 1:** Compounds affecting nerve regrowth in the fin removal assay. Compound name, presumptive action and previous studies about the role of the compound target in axonal/ nerve regeneration including references. Compounds tested by laser nerve transection are marked in the last column. Blue color highlights compounds whose presumptive action was expected to impair nerve regeneration based on previous reports. Red color highlights compounds whose presumptive action had been shown to have opposite effects on nerve regeneration. Compounds whose presumptive action has not been shown to control nerve regeneration are marked in white (compounds with no known targets) and yellow (with known targets).

Compound name	Presumptive action	Role of the compound target in axonal/ nerve regeneration	Reference	Tested by laser nerve transection
SB 202190	p38 MAPK inhibitor	Impaired regeneration in p38 knockout mice after crush injury.	[26]	
SB 203580	p38 MAPK inhibitor			
SP-600125	JNK inhibitor	JNK inhibitors delayed functional recovery/ delayed sensory nerve innervation to the skin after sciatic nerve transection in mice.	[27]	
U-0126	MEK1/2 inhibitor	Erk1/2 activation promoted robust retinal ganglion cells neuroprotection after optic nerve injury.	[28]	
Go6976	inhibitor of PKC, TrkA/B, TrkB, JAK2/3	NGF enhanced axonal regeneration *in vitro* and *in vivo*.	[29]	
pifithrin	p53 inhibitor	Pharmacological enhancement of the MDM2/p53-IGF1R axis enhanced axonal sprouting as well as functional recovery after spinal cord injury. Significant impairment in locomotor recovery in p53 KO versus WT mice spinal cord dorsal hemisection injury model.	1.[30] 2.[31]	
roscovitine	CDK 2, 5, 7, 9 inhibitor	Roscovitine inhibited regeneration of facial nerve axons after crush injury in rats. Schwann cells migrated less after roscovitine treatment *in vitro* and DRG neurites grow less in co-culture with Schwann cells.	1.[32] 2.[33]	
9-cis retinoic acid	retinoid X receptor agonist	Retinoic acid improved regeneration after nerve transection in rats.	[34]	AM580 was tested
AM-580	retinoic acid receptor agonist			x
dexamethasone	corticosteroid	Dexamethasone enhanced functional recovery after nerve crush injury in rats.	[35]	x
verapamil	L-type calcium channel inhibitor	Calcium channel inhibitor Nimodipin improved regeneration after nerve crush injury. Calcium channel inhibitor Nifedipin improved regeneration after nerve crush injury.	1.[36] 2.[37]	x
C2 dihydroceramide	inactive			
tyrphostin 1	inactive			
10-hydroxycampto-thecin (10-HCT)	topoisomerase I inhibitor	not known		x
9(S)-HpODE	fatty acid	not known		
anandamide (22:4,n-6)	cannabinoid ligand	not known		x
HA-1004	inhibitor of cyclic nucleotide-dependent protein kinases, e.g. PKC	*in vivo* effects not known (no effect of HA-1004 *in vitro* on PNS regeneration)	[38]	x
lavendustin A	EGFR inhibitor	not known		x
MnTBAP	SOD mimetic	not known		x
phenamil	TRPP3 channel inhibitor	not known		x
prostaglandin D2	prostaglandin	not known		x

### Compound validation using a laser based nerve transection assay

**Fig 4:**
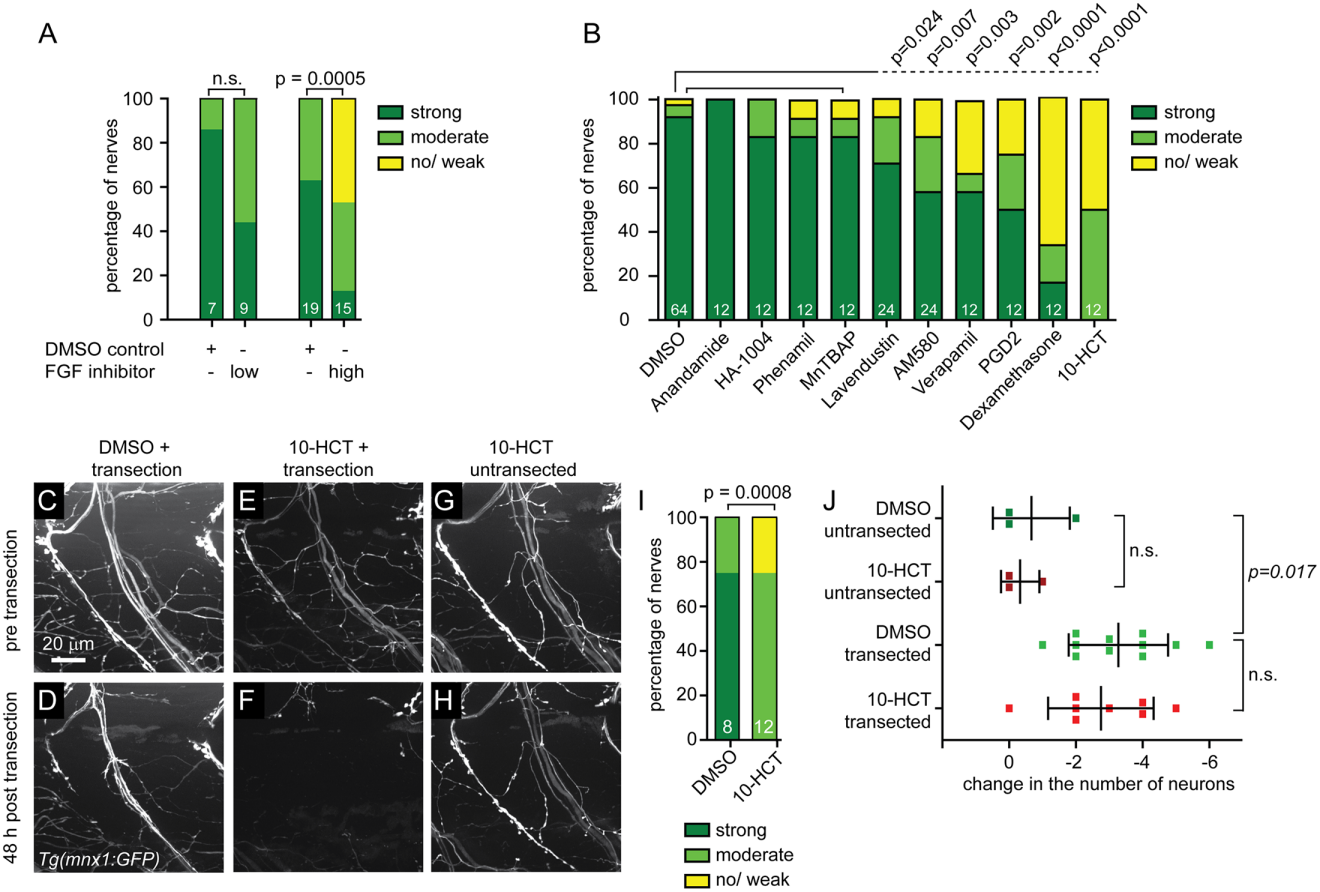
FGF inhibitor and six additional compounds that impair nerve regeneration after fin removal also impair regeneration after laser nerve transection. (A) Ventral nerves of *Tg(mnx1*:*GFP)* transgenic larvae were laser transected and treated with DMSO for control, or treated with a low (17μM) or high (34μM) dose of the FGF inhibitor SU5402. Regeneration was scored 48 hours later and the graphical representation of the extent of nerve regeneration (no/ weak, moderate, strong regeneration) is shown, demonstrating that the FGF inhibitor SU5402 significantly and dose-dependently impairs nerve regeneration. (B) Quantification of dorsal nerve regeneration in *Tg(isl1*:*GFP)* transgenic larvae measuring the extent of dorsal nerve regeneration (no/ weak, moderate, strong regeneration) for controls (0.5% DMSO) and all tested compounds. Post nerve transection exposure to 100μM anandamide (cannabinoid agonist), 25μg/ml HA-1004 (PKC inhibitor), 10μg/ml phenamil (TRPP3 channel blocker), and 25μg/ml MnTBAP (SOD mimetic) did not significantly impair nerve regeneration. However, 25μg/ml lavendustin (EGFR inhibitor), 4μM AM-580 (RA receptor agonist), 10μg/ml verapamil (calcium channel blocker), 5μM PGD2, 25μg/ml dexamethasone (corticosteroid), and 250μM 10-HCT significantly impaired nerve regeneration. (C-I) Ventral nerves of *Tg(mnx1*:*GFP)* transgenic larvae treated with DMSO for control (C, D), or with 125μM of the topoisomerase I inhibitor 10-HCT (E-H) before laser nerve transection (C, E, G) and 48 hours later (D, F, H). Control DMSO-treated larva showing normal morphology of the ventral nerve before transection (C), and robust nerve regeneration after 48 hours (D). Transected nerves treated with 10-HCT showed normal morphology before transection (E), but failed to regenerate after injury (F). In contrast, an untransected ventral nerve treated with 10-HCT showed normal morphology at all times (G,H). Graphical representation of the results, showing that 10-HCT significantly impaired nerve regeneration (I). (J) Change in the number of GFP-labeled neuronal cell bodies in *Tg(isl1*:*GFP)* transgenic larvae between day 5 and day 7, treated with DMSO for control or with 125μM 10-HCT. Dorsal nerves were either left untransected or laser transected at day 5. On average, 26 neurons were labeled per segment. There was no difference in the change of the neuronal cell body number between DMSO and 10-HCT treated larvae when left untransected or after nerve transection, suggesting that 10-HCT did not reduce neuronal survival. However, following laser nerve transection in both DMSO and 10-HCT treated larvae, the number of neurons decreased on average by 3 neurons, suggesting that a subset of neurons with transected axons died.

We next asked whether the compounds that impaired regeneration primarily inhibited wound healing or acted more directly on pathways that stimulate peripheral nerve regeneration. To distinguish between these possibilities, we used a laser to directly transect peripheral nerves, thereby minimizing collateral damage to other tissues [17, 39]. In brief, this well-established assay utilizes a dye laser (MicroPoint, Andor Technology) to transect the ventral or the dorsal nerve branch of individual, GFP positive motor nerves within the first ~20 μm of their peripheral trajectory [15–17]. We have previously shown that within 48 hours post transection injured motor axons exhibit robust regrowth towards their original targets resulting in functional regeneration [15–17]. Importantly, larvae were exposed to the compounds from 4 hours before transection until 48 hours post nerve transection, when axonal regeneration was scored (Fig 3). We tested 7 of the 8 compounds that affect known targets and whose presumptive actions were previously not known to affect peripheral nerve regeneration, as well as 3 of the 4 compounds that impaired regeneration but the presumptive action of the compound had previously been reported to promote regeneration. As a positive control, we tested the FGF inhibitor SU5402 and found that similar to impairing nerve regrowth following pectoral fin removal (Fig 2G–2J), SU5402 dose-dependently inhibited nerve regeneration following laser nerve transection (Fig 4A).

Using this assay, four of the ten tested compounds, the cannabinoid agonist anandamide, the inhibitor of cyclic nucleotide-dependent protein kinases HA-1004, the TRPP3 channel inhibitor phenamil, and the SOD mimetic MnTBAP failed to exhibit any effects on nerve regeneration (Fig 4B), suggesting that instead of targeting nerve specific regeneration pathways, they primarily affected tissue regeneration. In contrast, six of the ten compounds appeared to selectively target nerve regeneration, including the EGFR inhibitor lavendustin, the glucocorticoid receptor agonist dexamethasone, the RA receptor agonist AM580, prostaglandin D2 (PGD2), the calcium channel blocker verapamil, and the topoisomerase I inhibitor 10-HCT (Fig 4B–4I). Thus, pre-screening a small compound library using the fin removal assay identified six compounds whose presumptive action was previously not known to inhibit peripheral nerve regeneration *in vivo*.

### Topoisomerase I inhibitor selectively causes death of denervated Schwann cells

**Fig 5:**
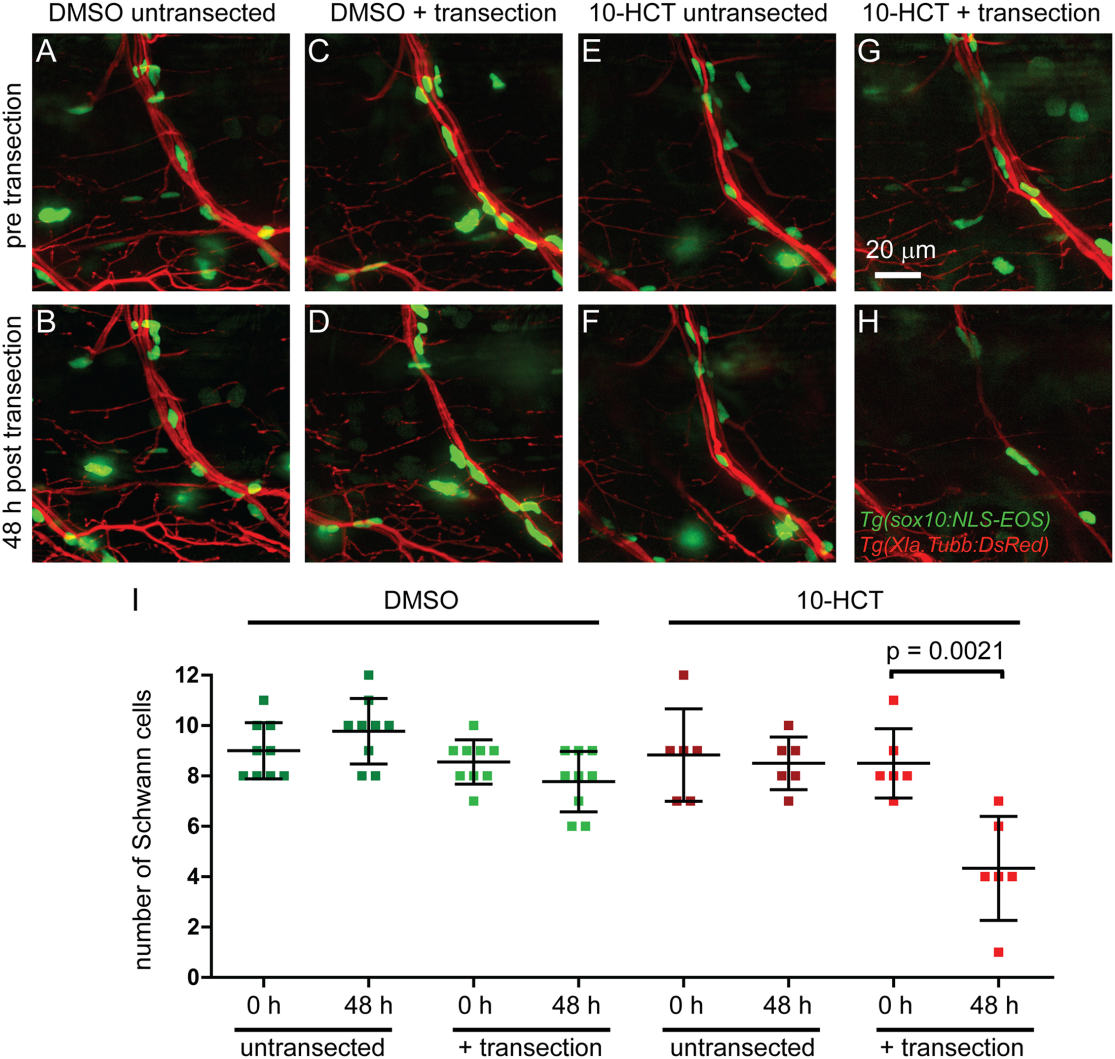
Topoisomerase I is required for survival of denervated Schwann cells following laser nerve transection. Ventral nerves of *Tg(Xla*.*Tubb*:*DsRed)*, *Tg(sox10*:*NLS-Eos)* double transgenic larvae treated with DMSO for control (A-D), or with the topoisomerase inhibitor 10-HCT (E-H); untransected controls (A,B,E,F) or laser nerve transected (C,D,G,H) at different time points: before transection (A,C,E,G) or 48 hours post transection (B,D,F,H). Graphical representation of the number of Schwann cells for the different conditions and time points (I). In DMSO controls, the number of Schwann cells was only non-significantly marginally reduced after laser nerve transection (C,D). Similarly, in untransected control nerves, 10-HCT did not reduce the number of Schwann cells, excluding the possibility that 10-HCT is simply toxic to Schwann cells. However, after laser nerve transection, the number of Schwann cells was significantly reduced, suggesting that 10-HCT specifically ablated denervated Schwann cells.

Of the six compounds whose presumptive action was previously not known to impair peripheral nerve regeneration, we decided to focus on the topoisomerase I inhibitor 10-HCT. Besides its canonical role in DNA replication [40], Topoisomerase I also promotes transcription [41], including transcription of long genes in neurons [42]. Both proliferation of glia cells as well as enhanced transcriptional activity in injured neurons and glia cells occur during regeneration [43–45], yet potential roles for topoisomerase I or its inhibitor 10-HCT in this process have not been reported. We first tested whether 10-HCT caused neuronal cell death thereby impairing axonal regeneration. Analysis of both uninjured and regenerating motor neurons did not reveal an overt loss of GFP positive motor neurons (Fig 4J). Genetic or acute ablation of Schwann cells in zebrafish severely impairs axonal regeneration [16, 18], and we therefore asked whether 10-HCT treatment affects the morphology or survival of Schwann cells critical for nerve regeneration [46]. We found that in untransected nerves, 10-HCT treatment did not affect the number of Schwann cells (Fig 5E, 5F and 5I). In contrast, 10-HCT treatment following laser nerve transected reduced the number of Schwann cells by ~50% (Fig 5G–5I). Thus, denervated Schwann cells are susceptible to 10-HCT treatment, consistent with the idea that topoisomerase I promotes Schwann cell survival after injury.

## Discussion

### Fin removal assay as a rapid and robust screening assay for nerve regeneration

Zebrafish adult and larvae have been used extensively to study fin regeneration, and in some instances regeneration of individual, unmyelinated sensory axons [47, 48]. In contrast to severing the tail fin, our assay using larval zebrafish focuses on the paired pectoral fins. In general, each spinal nerve of the zebrafish larva consists of ~60 axons and is associated with myelinating Schwann cells [16]. The pectoral fins are innervated by the anterior-most spinal nerves which show defined innervation patterns and consist of at least 30 axons [19, 20, 49]. We developed a simple yet robust fin removal assay that enabled us to perform the first whole organism small molecule screen to identify pathways that promote vertebrate nerve regeneration. Several lines of evidence support the validity of the fin removal assay. First, we show that nerve regrowth following an amputation-induced nerve transection exhibits key features of vertebrate peripheral nerve regeneration as both require Schwann cells and FGF signaling (Figs 2 and 4) [16, 18]. Second, 7 of the 21 (33%) compounds identified in the fin removal assay inhibit targets previously shown to promote nerve regeneration *in vivo* (Table 1). For example, the fin removal assay identified four inhibitors of MAPK signaling, including a JNK inhibitor, a MEK inhibitor, and two p38 inhibitors (Table 1). Previous studies have shown that MAPK signaling pathways are conserved and crucial regulators of peripheral nerve regeneration [26, 27, 50–53]. Third, we confirmed that the majority (6 of 10, 60%) of the compounds that inhibited axon regeneration in the fin removal assay also impaired peripheral nerve regeneration in the lower-throughput but better-established laser nerve transection assay (Fig 4). However, during the second pass of the screen, we failed to identify a singly effective compound in three out of 15 compound pools (20%), which reduced nerve regrowth in the first pass of the screen. This reflects either false positive results or an effect of two or more compounds acting together to impair regeneration. Using laser nerve transections as the gold standard to test peripheral nerve regeneration, the rate of false positive results during the second pass of the fin removal assay was also relatively high (4 of 10, 40%). Thus, the fin removal assay is a powerful and robust screening method for identifying targets required for peripheral nerve regeneration. Given the high false positive rate, the fin removal assay should always be combined with a second independent method to study nerve regeneration. The assay can easily be modified, for example by looking at an earlier time point, when controls have not reformed the ring-like network at the fin base, we would be able to identify compounds which speed up regeneration. Combined with targeted genome editing to generate genetic mutants, it presents a powerful platform to identify genes that promote peripheral nerve regeneration.

### Topoisomerase I inhibition selectively reduces integrity of denervated Schwann cell

We identified six compounds that impair peripheral nerve regeneration in both the fin amputation and laser axotomy assay. The glucocorticoid dexamethasone, as well as calcium channel inhibition and retinoid acid agonism have previously been reported to promote rather than to inhibit axonal regeneration [34–37]. In contrast, we find that the three compounds, dexamethasone as well as the calcium channel inhibitor verapamil and the retinoic acid agonist AM-580 inhibit rather than promote nerve regeneration. These discrepancies might be due to differences in compound dosage, specific compound used to target a pathway, and different treatment times and periods. For the remaining three compounds/targets lavendustin/EGFR, 10-HCT/topoisomerase I and PGD2 we provide the first evidence for effects on peripheral nerve regeneration *in vivo*. While these compounds might directly affect axonal outgrowth, it is also possible that these compounds primarily act on Schwann cells. For example, EGFR signaling has been shown to promote Schwann cell migration and proliferation [54]. Similarly, the prostanoid PGD2 has been shown activate the G-protein-coupled receptor Gpr44 expressed on Schwann cells [55].

Finally, our data reveal that the topoisomerase I inhibitor 10-HCT decreases survival of Schwann cells associated with injured but not intact peripheral nerves (Fig 5). Topoisomerase I inhibitors including 10-HCT are clinically used as chemotherapeutic agents in cancer therapy by inducing DNA damage and apoptosis of proliferating cells [56, 57]. Thus, 10-HCT might simply cause apoptosis of proliferating Schwann cells. However, in control treated animals we did not observe an increase in Schwann cell number following laser nerve transection (Fig 5), in line with previous findings that the early stages of peripheral nerve regeneration in zebrafish occur independently of Schwann cells proliferation [16]. In addition to its canonical role in DNA replication, topoisomerase I is also required for gene expression [58], and was found more recently to control transcription of long genes [42]. Importantly, following nerve injury, Schwann cells shed their myelin and de-differentiate to a more neural crest-/ stem cell-like state that allows them proliferate and migrate [59]. Although we cannot exclude that 10-HCT has a topoisomerase I-independent effect on regeneration, one attractive hypothesis is that topoisomerase I promotes peripheral nerve regeneration by regulating gene transcription specifically in de-differentiated Schwann cells (Fig 5). Independent of its effects on Schwann cells, topoisomerase I inhibition might also affect axonal regrowth directly. Future studies are required to determine the precise mechanism by which 10-HCT/ topoisomerase I inhibition affects peripheral nerve regeneration. Independent of the precise underlying mechanism, we developed a rapid assay that combined with a whole organism small molecule screen identified several compounds previously not known to affect peripheral nerve regeneration *in vivo*.

## Materials and methods

### Zebrafish care and strains

Protocols and procedures involving zebrafish are in compliance with the University of Pennsylvania Institutional Animal Care and Use Committee (IACUC) regulations, and IACUC specifically approved this study. Adult zebrafish were kept in tanks in groups of 20 to 60 fish and fed with paramecia or dry flake food. They were exposed to a regular light-dark cycle. Water was changed daily and water quality was tested daily. Embryos were generated by natural mating as described [60]. All embryos were raised at 28°C. The *Tg(mnx1*:*GFP)*^*ml2*^ (Flanagan [61], *Tg(isl1*:*GFP)*, [62], and *Tg(Xla*.*Tubb*:*DsRed)*^*zf148*^ [63] transgenic lines were used to label spinal motor nerves. The *Tg(sox10*:*NLS-Eos)*^*w18*^ [64] transgenic line was used to label Schwann cell nuclei. All experimental and control animals were randomly distributed into control and experimental group.

### Immunohistochemistry

Embryos were fixed in 4% paraformaldehyde with 1% DMSO in 0.1 M phosphate buffer, pH 7.4, then dehydrated in methanol and permeabilized for 30 min. in acetone at -20°C, and rehydrated with incubation buffer (0.2% BSA, 0.5% Triton X 100 in 0.1 M phosphate buffer, pH 7.4). The primary antibody anti-SV2 was used at 1:50 (Developmental Studies Hybridoma Bank [DSHB]). Embryos were washed at least three times in incubation buffer before adding the secondary antibody conjugated with Alexa Fluor 488 (1:400, Life Technologies). Antibody incubations were performed for 4 hours at room temperature or overnight at 4°C. Embryos were mounted in Vectashield mounting medium (Vector laboratories), and samples were viewed and documented as described below.

### Fin removal assay

5-day-old *Tg(mnx1*:*GFP)* transgenic larval zebrafish were anesthetized using tricaine, and larvae were mounted on 1.5% agarose plates molded with individual wells for each larva. Larvae were laid on their sides and covered by 0.7% low-melt agarose containing 0.01% tricaine. Mounted larvae were then covered by E3 medium containing tricaine. We used a stereo microscope to view the procedure, and tweezers #5 (World Precision Instruments) to handle the pectoral fins. For each fin amputation, the fin was lifted away from the larval body and grasped at its base. Then we pulled the fin out and visually confirmed that the entire fin including the base was removed. Four fish per condition were placed in a 48-well plate containing 500 μl E3 and treated with 0.5% DMSO, compound pools (each compound diluted 1:1,000) in 0.5% DMSO, or individual compounds in 0.5% DMSO. 24 hours after fin removal, larvae were mounted in 1.2% agarose, and regrown fin nerves were imaged on a spinning disc confocal microscope (Olympus; see below).

### Small molecule screen

We used the ICCB Known Bioactives library (Enzo). Compounds were added to E3 medium in which larval zebrafish were kept and placed in a 28°C incubator. All larvae tested were drug naive. To determine toxicity of compounds, we tested all compounds individually on at least three 5-day-old larval zebrafish for 48 hours at a 1:1,000 dilution (for stock concentrations see S1 Table). Those compounds inducing tissue necrosis, cardiac arrest, alteration of body morphology, or strongly reduced responsiveness to touch after 12, 24 or 48 hours were called toxic and excluded from further testing. Non-toxic compounds were assigned to 69 distinct pools and tested for their effects on nerve regrowth using the fin removal assay (first-pass). Usually, each pool was tested on four larvae, if the result was not clear (regeneration categories could not easily be determined), the test was repeated. Pools which caused impaired regeneration in at least half of the tested larvae (usually 2 out of 4) were called "hits". If only three larvae survived the treatment but showed normal regeneration, the pool was defined as not being a "hit". We called regeneration abnormal if it was either category 0 or 1 regeneration or if regrowth of the ring-like nerve network at the fin base was severely disorganized. All the individual compounds in the 15 "hit" pools were tested individually in the second-pass using the fin removal assay. Before testing the individual compounds, the highest tolerated concentration was determined. For this, dilutions of 1:100 to 1:1,000 were tested on 5-day-old larvae. 21 compounds caused abnormal nerve regrowth at the fin base. Each compound pool had a number code and each compound in the pool a letter code. Identity of pools and compounds was only revealed after scoring regeneration, reducing subjective bias when scoring regeneration. The highest tolerated dose of 10 of those compounds was tested using laser nerve transection (see below). Control and experimental groups were treated in the same order as regeneration was assessed to ensure approximately equal treatment duration. Treatment and assessment were done for one group after the other.

### Nerve transection by laser nerve transection

Transection of dorsal peripheral nerves was performed as previously described [15, 17]. For scoring of dorsal and ventral nerve regrowth following laser nerve transection, we used three categories (no/weak, moderate, strong) as introduced in [15] and adapted this scoring system to dorsal nerves. Larvae were exposed to the compounds for at least 4 hours before and then for 48 hours after nerve transection when regeneration was assessed.

### Imaging, image processing and data analysis

Live larvae or fixed and stained embryos mounted in Vectashield (Vector laboratories) were imaged using a spinning disk confocal microscope (Olympus). Maximum intensity projection images of z-stacks were created using Slidebook (3i) software. Live embryos were mounted in 1.2% agarose in E3 and anesthetized in 0.022% tricaine. Confocal images were further processed using the Image J software package (NIH). Image manipulations included adjustment of brightness, contrast, gamma-value, and background subtraction. Manipulations were always applied to the entire image and to all images in one experiment, ensuring that the content of the image wasn't altered. Images were exported and further processed in Photoshop CS4 and final versions of the figures for the manuscript were prepared using Illustrator CS4 and Photoshop CS4 (Adobe). P-values were calculated using the Fisher exact test for categorical outcomes using a Graph Pad web tool (GraphPad). Graphs were generated using Prism 5 (GraphPad).

## Supporting information

S1 ARRIVE checklist Completed ARRIVE guidelines checklist. (PDF)

S1 Table Table showing all compounds of the ICCB Known Bioactives library (Enzo) including action, CAS#, and stock concentration. The compounds which were pooled and tested in the fin removal assay are on top and toxic compounds are below. Compound pool composition, effect of compound pools on fin removal assay, and which compound (if any) of the pool impaired nerve regeneration in the fin removal assay.
(PDF)
