# Supplementary material for: A small molecule screen identifies in vivo modulators of peripheral nerve regeneration in zebrafish
Source: PLoS One. 2017 Jun 2;12(6):e0178854. doi: 10.1371/journal.pone.0178854 (PMC5456414; doi:10.1371/journal.pone.0178854)
Supplement: S1 Table — The compounds which were pooled and tested in the fin removal assay are on top and toxic compounds are below. Compound pool composition, effect of compound pools on fin removal assay, and which compound (if any) of the pool impaired nerve regeneration in the fin removal assay. (PDF) [file pone.0178854.s002.pdf]

pool#	compound name	action	stock conc	CAS#	effect of pool in fin removal assay	individual compounds impairing regrowth in fin removal assay (dilution of stock tested)
1	C16 Ceramide	Activates PKC zeta	1mM	24696-26-2		
1	12(S)-HPETE	Fatty acid hydroperoxide	0.1mM	71774-10-2	n=3/4 normal	
1	15d-Prostaglandin J2	Bioactive prostaglandin	1mM	87893-55-8		
1	4-Oxatetradecanoic acid	Myristic acid analog	1mM			
1	C8 Ceramine	Ceramide analog. Apoptosis inducer	1mM			
2	1-Octadecyl-2-methylglycero-3 PC	Inhibits PI-specific PLC	1mM	77286-66-9		none of the individual compounds caused abnormal regrowth
2	12-Methoxydodecanoic acid	Myristic acid analog	1mM	92169-28-3	n=3/4 misguided	
2	8,9-Epoxyeicosatrienoic acid	Bioactive arachidonic acid metabolite	0.1mM			
2	AGC	Negative control for AGGC and AFC	1mM			
2	Arachidonoyl-PAF	PAF precursor	1mM	86288-11-1		
3	15-Ketoeicosatetraenoic acid	Bioactive arachidonic acid metabolite	0.1mM	81416-72-0		
3	1-Oleoyl-2-acetyl-glycerol	PKC activator	1mM	86390-77-4	n=8/8 normal	
3	5(S)-HETE	Bioactive arachidonic acid metabolite	0.1mM	70608-72-9		
3	8-EPI-prostaglandin F2α	Thromboxane TP receptor agonist	1mM	27415-26-5		
3	AGGC	ICMT inhibitor	1mM	139332-94-8		
4	(R)-Methanandamide	Cannabinoid CB1 receptor agonist	1mM	150314-39-3		
4	13(S)-HODE	Bioactive linoleic acid metabolite	0.1mM	29623-28-7	n=7/7 normal	
4	16,16-Dimethyl-prostaglandin E2	Prostaglandin EP receptor agonist	1mM	39746-25-3		
4	1-Stearoyl-2-linoleoyl-glycerol	PKC activator	1mM			
4	BML-190	Cannabinoid CB1 inverse agonist	1mM			
5	13(S)-HPODE	Fatty acid hydroperoxide	0.1mM	33964-75-9		
5	5(S)-HPETE	Fatty acid hydroperoxide	0.1mM	71774-08-8	n=11/12 normal	
5	9(S)-HODE	Bioactive linoleic acid metabolite	0.1mM	73543-67-6		
5	AM-251	Cannabinoid CB1 receptor antagonist	1mM			
5	C2 Ceramide	Apoptosis inducer	1mM	3102-57-6		
6	17-Octadecynoic acid	Inhibits fatty acid omega oxidation	1mM	34450-18-5		
6	1-Stearoyl-2-arachidonoyl-glycerol	PKC activator	1mM	65914-84-3	n=8/11 abnormal<br>n=5/11 misguided and n=3/11 reduced regrowth	
6	5,6-Epoxyeicosatrienoic acid	Bioactive arachidonic acid metabolite	0.1mM			
6	9(S)-HPODE	Fatty acid hydroperoxide	0.1mM	29774-12-7		9(S)-HPODE (1:100)
6	AM-580	Retinoid RAR agonist	1mM	102121-60-8		AM-580 (1:100)
7	1,2-Didecanoyl-glycerol (10:0)	Activates PKC	1mM	82950-64-9		

7	13,14-Dihydro-prostaglandin E1	Bioactive prostaglandin	1mM	19313-28-1	n= 5/8 abnormal n=3/8 misguided and n=2/8 reduced regrowth	
7	17-Phenyl-trinor-prostaglandin E2	Prostaglandin EP1 receptor agonist	1mM	38315-43-4		
7	5,8,11,14-Eicosatetraynoic acid	Cyclooxygenase & lipoxygenase inhibitor	1mM	1191-85-1		
7	C2 Dihydroceramide	Negative control for C2 ceramide	1mM			C2 Dihydroceramide (1:100)
8	1,2-Dioctanoyl-sn-glycerol	Activates PKC	1mM	75685-80-2		
8	13-cis Retinoic acid	Retinoid receptor ligand	1mM	4759-48-2	n=4/4 normal	
8	1-Acyl-PAF	PAF agonist	1mM			
8	9,10-Octadecenoamide	Endogenous sleep inducing lipid	1mM			
8	Anandamide (18:2,n-6)	Cannabinoid receptor agonist	1mM			
9	1,2-Dioleoyl-glycerol (18:1)	Activates PKC	1mM	3738-74-7		
9	5,8,11-Eicosatriynoic acid	Lipoxygenase inhibitor	1mM	13488-22-7	n=4/4 normal	
9	9alpha,11beta-Prostaglandin F2	Bioactive prostaglandin	1mM	38432-87-0		
9	Anandamide (20:3,n-6)	Cannabinoid receptor agonist	1mM			
9	C8 Dihydroceramide	Negative control for C8 ceramide	1mM			
10	13-Ketooctadecadienoic acid	Bioactive linoleic acid metabolite	0.1mM			
10	1-Hexadecyl-2-arachidonoyl-glycerol	DAG analog	1mM		n=3/3 normal	
10	2-Arachidonoylglycerol	Cannabinoid CB1 receptor agonist	1mM	53847-30-6		
10	5-Ketoeicosatetraenoic acid	5-KETE receptor (R527) agonist	0.1mM			
10	9beta,11alpha-Prostaglandin F2	Bioactive prostaglandin	1mM	4510-16-1		
11	11,12-Epoxyeicosatrienoic acid	Bioactive arachidonic acid metabolite	0.1mM			
11	14,15-Epoxyeicosatrienoic acid	Bioactive arachidonic acid metabolite	0.1mM		n=8/8 normal	
11	1-Hexadecyl-2-methylglycero-3 PC	PAF receptor agonist	1mM			
11	Anandamide (20:4, n-6)	Cannabinoid receptor agonist	1mM	94421-68-8		
11	Carbacyclin	PPAR delta agonist	1mM	69552-46-1		
12	12(R)-HETE	Bioactive arachidonic acid metabolite	0.1mM	82337-46-0		
12	2-Fluoropalmitic acid	Protein palmitoylation inhibitor	1mM	89270-22-4	n=4/8 abnormal	
12	9-cis Retinoic acid	Retinoid RXR agonist	1mM	5300-03-8	n=3/8 misguided and n=1/8 reduced regrowth	9-cis Retinoic acid (n=4/4 reduced regrowth; 1:300) Anandamide (22:4,n-6) (n=4/4 reduced regrowth; 1:100)
12	Anandamide (22:4,n-6)	Cannabinoid receptor agonist	1mM			
12	Ciglitazone	PPAR gamma agonist	1mM	74772-77-3		
13	15(S)-HETE	Bioactive arachidonic acid metabolite	0.1mM	54845-95-3		
13	1-Hexadecyl-2-O-acetyl-glycerol	Blocks DAG activation of PKC	1mM	77133-35-8	n=5/5 normal	
13	2-Hydroxymyristic acid	Protein myristoylation inhibitor	1mM	2507-55-3		

13	6-Keto-prostaglandin F1α	Bioactive prostaglandin	1mM	58962-34-8	
13	Dimethyloxaloylglycine	Prolyl-4-hydroxylase inhibitor	1mM	89464-63-1	
14	12(S)-HETE	Bioactive arachidonic acid metabolite	0.1mM	54397-83-0	
14	15(S)-HPETE	Fatty acid hydroperoxide	0.1mM	70981-96-3	n=4/4 normal
14	1-Hexadecyl-2-O-methyl-glycerol	Blocks DAG activation of PKC	1mM		
14	Arachidonamide	Bioactive arachidonic acid metabolite	1mM		
14	Clofibrate	PPAR alpha agonist	1mM	637-07-0	
15	4-Hydroxyphenylretinamide	Retinoid receptor agonist / apoptosis inducer	1mM	6546-68-6	
15	7,7-Dimethyleicosadienoic acid	PLA2 inhibitor	1mM	89560-01-0	n=4/4 normal
15	Adrenic acid (22:4, n-6)	Polyunsaturated fatty acid	1mM	28874-58-0	
15	Arachidonic acid (20:4, n-6)	Polyunsaturated fatty acid	1mM	506-32-1	
15	Cloprostenol Na	Prostaglandin FP receptor agonist	1mM	55028-72-3	
15	C-PAF	PAF receptor agonist	1mM	91575-58-5	
16	Docosatrienoic acid (22:3 n-3)	Polyunsaturated fatty acid	1mM	28845-86-5	
16	Leukotoxin B (12,13-EODE)	Bioactive linoleic acid metabolite	0.1mM		n=4/4 normal
16	LY-171883	Leukotriene D4 receptor antagonist	1mM	88107-10-2	
16	N-Acetyl-leukotriene E4	Bioactive arachidonic acid metabolite	0.1mM	80115-95-3	
16	Prostaglandin B1	Bioactive prostaglandin	1mM	13345-51-2	
17	Delta 12-Prostaglandin J2	Bioactive prostaglandin	1mM	87893-54-7	
17	Eicosa-5,8-dienoic acid (20:2 n-12)	Polyunsaturated fatty acid	1mM		n=7/7 normal
17	Leukotriene B4	Leukotriene B4 receptor agonist	0.1mM	71160-24-2	
17	Lyso-PAF C16	Inactive PAF metabolite	1mM	52691-62-0	
17	4-Aminopyridine	Potassium channels	5mg/mL	504-24-5	
18	Dihomo-gamma-linolenic acid	Polyunsaturated fatty acid	1mM	1783-84-2	
18	N-Arachidonoylglycine	FAAH inhibitor	1mM		n=4/4 normal
18	Prostaglandin B2	Bioactive prostaglandin	1mM	13367-85-6	
18	S-Farnesyl-L-cysteine methyl ester	MDR ATPase activator	1mM		
18	5-Hydroxydecanoate	Potassium channels	5mg/mL	624-00-0	
19	Eicosadienoic acid (20:2 n-6)	Polyunsaturated fatty acid	1mM	2091-39-6	
19	Leukotriene C4	CysLT receptor agonist	0.1mM	72025-60-6	n=2/4 abnormal
19	Lysophosphatidic acid	LPA receptor antagonist	1mM	22556-62-3	n=1/4 misguided and n=1/4 reduced regrowth
19	N-Linoleoylglycine	FAAH inhibitor	1mM		

19	Prostaglandin D2	Prostaglandin DP receptor agonist	1mM	41598-07-6		Prostaglandin D2 (1:100)
20	Dihydrosphingosine	Apoptosis inducer	1mM			
20	Eicosapentaenoic acid (20:5 n-3)	Polyunsaturated fatty acid	1mM	10417-94-4	n=7/7 normal	
20	Leukotriene D4	CysLT receptor agonist	0.1mM	73836-78-9		
20	MAPP, D-erythro	Ceramidase inhibitor	1mM			
20	Sphingosine	PKC inhibitor	1mM	123-78-4		
21	Diindolylmethane	AHR agonist	1mM	1968-05-4		
21	PAF C16	PAF receptor agonist	1mM	74389-68-7	n=4/4 normal	
21	Prostaglandin E1	Prostaglandin EP receptor agonist	1mM	745-65-3		
21	SQ-29548	Thromboxane A2 antagonist	1mM	98299-61-7		
21	AM 92016	Potassium channels	5mg/mL	178894-81-0		
22	Eicosatrienoic acid (20:3 n-3)	Polyunsaturated fatty acid	1mM	2091-27-2		
22	Leukotriene E4	CysLT1 receptor agonist	0.1mM	75715-89-8	n=4/4 normal	
22	MAPP, L-erythro	Negative control for D-erythro-MAPP	1mM			
22	PAF C18	PAF receptor agonist	1mM	79549-26-1		
22	Prostaglandin E2	Prostaglandin EP receptor agonist	1mM	363-24-6		
23	DL-Dihydrosphingosine	Sphingosine kinase inhibitor	1mM	73938-69-9		
23	Enantio-PAF C16	Negative control for PAF	1mM	117985-57-6	n=4/4 normal	
23	Linoleamide	Bioactive linoleic acid metabolite	1mM			
23	Tetrahydrocannabinol-7-oic acid	PPAR gamma agonist	1mM	39690-06-7		
23	Amantidine	Misc. channels	5mg/mL	665-66-7		
24	DL-PDMP	Glucosylceramide synthase inhibitor	1mM	73257-80-4		
24	Mead acid (20:3 n-9)	Polyunsaturated fatty acid	1mM	20590-32-3	n=4/4 normal	
24	PAF C18:1	PAF receptor agonist	1mM	86288-90-1		
24	Prostaglandin F1α	Prostaglandin FP receptor agonist	1mM	745-62-0		
24	Amiloride	Calcium channels	5mg/mL	2016-88-8		
25	Farnesylthioacetic acid	Carboxymethylation inhibitor	1mM	135784-48-4		
25	Linoleic acid	Polyunsaturated fatty acid	1mM	60-33-3	n=4/4 normal	
25	Mead ethanolamide	Cannabinoid receptor agonist	1mM	169232-04-6		
25	Palmitylethanolamide	Cannabinoid CB2 receptor agonist	1mM	544-31-0		
25	Prostaglandin F2α	Prostaglandin FP receptor agonist	1mM	551-11-1		
26	DL-PPMP	Glucosylceramide synthase inhibitor	1mM			

26	Fluprostenol	Prostaglandin FP receptor agonist	1mM	40666-16-8	n=4/4 normal
26	Linolenic acid (18:3 n-3)	Polyunsaturated fatty acid	1mM	463-40-1	
26	Methoprene acid	Retinoid RXR agonist	1mM		
26	U-46619	Thromboxane TP receptor agonist	1mM	56985-40-1	
27	Docosahexaenoic acid (22:6 n-3)	Polyunsaturated fatty acid	1mM	6217-54-5	
27	γ-Linolenic acid (18:3 n-6)	Πολψυνσατυρατεδ φαττψ αχιδ	1mM	506-26-3	n=4/4 normal
27	Phosphatidic acid, dipalmitoyl	Activates MAP kinase cascade	1mM	71065-87-7	
27	Prostaglandin I2 Na	Prostaglandin IP receptor agonist	1mM	61849-14-7	
27	U-75302	Leukotriene B4 receptor antagonist	0.1mM	119477-85-9	
28	Lipoxin A4	Bioactive arachidonic acid metabolite	0.1mM	89663-86-5	
28	Misoprostol, free acid	Prostaglandin EP receptor agonist	1mM	59122-46-2	n=4/4 normal
28	Prostaglandin A1	Bioactive prostaglandin	1mM	14152-28-4	
28	Prostaglandin J2	Bioactive prostaglandin	1mM	60203-57-8	
28	WIN 55,212-2	Cannabinoid CB1/CB2 receptor agonist	1mM	131543-23-2	
29	Docosapentaenoic acid	Polyunsaturated fatty acid	1mM	2234-74-4	
29	Leukotoxin A (9,10-EODE)	Bioactive linoleic acid metabolite	0.1mM		n=3/3 normal
29	L-NASPA	LPA agonist / antagonist	1mM		
29	N,N-Dimethylsphingosine	Sphingosine kinase inhibitor	1mM	119567-63-4	
29	Prostaglandin A2	Bioactive prostaglandin	1mM	13345-50-1	
30	Retinoic acid, all trans	Retinoid RAR agonist	1mM	302-79-4	
30	WY-14643	PPAR alpha agonist	1mM	50892-23-4	n=3/3 normal
30	Benzamil	Calcium channels	5mg/mL	2898-76-2	
30	Loperamide	Calcium channels	5mg/mL	34552-83-5	
30	Pinacidil	Potassium channels	5mg/mL	85371-64-8	
31	Methoxy verapamil	Calcium channels	5mg/mL	16662-47-8	
31	Procainamide	Sodium channels	5mg/mL	614-39-1	n=4/4 normal
31	TMB-8	Intracellular calcium	5mg/mL	53464-72-5	
31	Thiorphan	Neutral endopeptidase inhibitor	5mg/mL	76721-89-6	
31	2-Methoxyantimycin A3	Bcl-2/Bcl-XL ligand induces apoptosis	5mg/mL		
32	Dantrolene	Intracellular calcium	5mg/mL	7261-97-4	
32	Minoxidil	Potassium channels	5mg/mL	38304-91-5	n=5/5 normal
32	Propafenone	Potassium channels	5mg/mL	54063-53-5	

32	Tolazamide	Potassium channels	5mg/mL	1156-19-0		
32	Z-Prolyl-prolinal	Prolyl endopeptidase inhibitor	5mg/mL	86925-97-5		
33	Quinidine	Sodium channels	5mg/mL	6151-40-2		
33	Tolbutamide	Potassium channels	5mg/mL	64-77-7	n=7/8 normal	
33	Okadaic acid	PP1 PP2A inhibitor	0.5mg/mL	78111-17-8		
33	Taxol = Paclitaxel	microtubule stabilizer	5mg/mL	33069-62-6		
33	Gingerol	Intracellular calcium	5mg/mL	23513-14-6		
34	Dichlorobenzamil	Calcium channels	5mg/mL	1166-01-4		
34	Glipizide	Potassium channels	5mg/mL	29094-61-9	n=7/8 normal	
34	PCO-400	Potassium channels	5mg/mL	121055-10-5		
34	NapSul-Ile-Trp-CHO	Cathepsin L inhibitor	5mg/mL			
34	Betulinic acid	induces mitochondrial permeability pore opening	5mg/mL	472-15-1		
35	Diltiazem	Calcium channels	5mg/mL	33286-22-5		
35	Glyburide	Potassium channels	5mg/mL	10238-21-8	n=4/4 normal	
35	Quinine	Potassium channels	5mg/mL	6119-47-7		
35	U-37883A	Potassium channels	5mg/mL	57568-80-6		
35	Deprenyl	inhibits glyceraldehyde-3-phosphate dehydrogenase	5mg/mL	14611-52-0		
36	Nifedipine	Calcium channels	5mg/mL	21829-25-4		
36	QX-314	Sodium channels	5mg/mL	21306-56-9	n=3/4 normal	
36	U-50488	Calcium channels	5mg/mL	67198-13-4		
36	Boc-GVV-CHO	Gamma secretase inhibitor	5mg/mL			
36	1400W	iNOS inhibitor	5mg/mL			
37	E-4031	Potassium channels	5mg/mL	113558-89-7		
37	Grayanotoxin III	Sodium channels	5mg/mL	4678-45-9	n=4/4 abnormal	
37	Phenamil	Sodium channels	5mg/mL	2038-35-9	n=2/4 misguided and n=2/4 reduced regrowth	Phenamil (1:500)
37	Verapamil	Calcium channels	5mg/mL	23313-68-0		Verapamil (1:500)
37	Bestatin	Aminopeptidase inhibitor	5mg/mL	65391-42-6		
38	Flecainide	Sodium channels	5mg/mL	54143-56-5		
38	IAA-94	Misc. channels	5mg/mL	54197-31-8	n=4/4 normal	
38	Ala-Ala-Phe-CMK	Tripeptidyl peptidase II inhibitor	5mg/mL			
38	Fumonisin B1	inhibits ceramide synthase	5mg/mL	116355-83-0		
38	Tanshinone IIA	AP-1 inhibitor	5mg/mL			

39	L-cis-Diltiazem	Calcium channels	5mg/mL			
39	Lidocaine	Sodium channels	5mg/mL	6108-05-0	n=8/8 normal	
39	Phenytoin	Sodum channels	5mg/mL	57-41-0		
39	YS035	Calcium channels	5mg/mL	33978-72-2		
39	Bromo-cAMP [8-Bromo-cAMP]	PKA activator	5mg/mL	76939-46-3		
40	Tetrandrine	Calcium channels	5mg/mL	518-34-3		
40	Ac-Leu-Leu-Nle-CHO	Calpain inhibitor	5mg/mL		n=4/4 abnormal	none of the individual compounds caused abnormal regrowth
40	Lycorine	inhibits TNFalpha production	5mg/mL	476-28-8	n=1/4 misguided and n=3/4 reduced regrowth	
40	A-3	kinase inhibitor	5mg/mL			
40	AG-1296	c-kit, FGF and PDGF kinase inhibitor	5mg/mL	146535-11-7		
41	TRIM	bNOS/iNOS inhibitor	5mg/mL	25371-96-4		
41	Acetyl (N)-S-farnesyl-L-cysteine	farnesylation inhibitor	5mg/mL	135304-07-3	n=8/8 normal	
41	Milrinone	phosphodiesterase (PDE3) inhibitor	5mg/mL	78415-72-2		
41	8-Methoxymethyl-IBMX	phosphodiesterase (PDE1) inhibitor	5mg/mL	78033-08-6		
41	Cimaterol	adrenoceptor agonist (beta)	5mg/mL	54239-37-1		
42	MDL-28170	Calpain inhibitor	5mg/mL	88191-84-8		
42	Actinomycin D	transcription inhibitor	5mg/mL	50-76-0	n=3/3 normal	
42	PRIMA-1	p53 reactivator	5mg/mL	5608-24-2		
42	B581	farnesyltransferase inhibitor	5mg/mL	149759-96-6		
42	CinnGEL 2Me	PTP1B inhibitor	5mg/mL			
43	Zaprinast	phosphodiesterase (PDE1) inhibitor	5mg/mL	37762-06-4		
43	Arvanil	Vaniloid receptor agonist	5mg/mL		n=3/3 normal	
43	BADGE	PPAR gamma antagonist	5mg/mL	1675-54-3		
43	Bromo-cGMP [8-Bromo-cGMP]	PKG activator	5mg/mL	51116-01-9		
43	Cirazoline	adrenoreceptor agonist (alpha)	5mg/mL	40600-13-3		
44	MG-132	Proteasome inhibitor	5mg/mL	133407-82-6		
44	Bumetanide	Na+K+Cl- cotransport inhibitor	5mg/mL	28395-03-1	n=4/4 normal	
44	5'-N-Ethylcarboxamidoadenosine (NECA)	adenosine receptor agonist	5mg/mL	78647-50-4		
44	Capsacin(E)	vanilloid receptor agonist	5mg/mL	404-86-4		
44	Clonidine	adrenoreceptor agonist (alpha)	5mg/mL	4205-91-8		
45	AG213 (Tyrphostin 47)	EGF-R tyrosine kinase inhibitor	5mg/mL	122520-86-9		
45	Aminobenzamide (3-ABA) [3-Aminobenzamide (3-ABA)]	ADP ribose polymerase, apoptosis inhibitor	5mg/mL	3544-24-9	n=4/4 normal	

45	BAPTA-AM	cell permeable Ca++ chelator	5mg/mL	139890-68-9		
45	BW-B 70C	5 lipoxygenase inhibitor	5mg/mL	134470-38-5		
45	Capsazepine	vanilloid receptor antagonist	5mg/mL	138977-28-3		
46	Bezafibrate	PPAR alpha agonist	5mg/mL	41859-67-0		
46	Siguazodan	phosphodiesterase (PDE3) inhibitor	5mg/mL	115344-47-3	n=6/7 normal	
46	AG-370	PDGF receptor kinase inhibitor	5mg/mL			
46	CA-074-Me	Cathepsin B inhibitor	5mg/mL			
46	Castanospermine	glucosidase inhibitor	5mg/mL	79831-76-8		
47	Cycloheximide-N-ethylethanoate	FKBP12 inhibitor	5mg/mL			
47	AG-490	JAK2 inhibitor	5mg/mL		n=4/4 normal	
47	Bongkrekic acid	ANT inhibitor	0.5mg/mL	11076-19-0		
47	SB-415286	GSK3 beta inhibitor	5mg/mL	264218-23-7		
47	Ascomycin (FK-520)	binds to FKBP inhibits calcineurin	5mg/mL	11011-38-4		
48	Anisomycin	MAP kinase activator	5mg/mL	22862-76-6		
48	Ro 20-1724	phosphodiesterase (PDE4) inhibitor	5mg/mL	29925-17-5	n=4/4 normal	
48	Aphidicolin	DNA polymerase inhibitor	5mg/mL	38966-21-1		
48	IB-MECA	Adenosine receptor agonist	5mg/mL	152918-18-8		
48	Cyclosporin A	calcineurin inhibitor	5mg/mL	59865-13-3		
49	Alamethicin	monovalent cation ionophore	5mg/mL	27061-78-5		
49	Calpeptin	Calpain inhibitor	5mg/mL		n=5/5 normal	
49	Calphostin C	PKC inhibitor	0.5mg/mL	121263-19-2		
49	Chelerythrine	PKC inhibitor	5mg/mL	3895-92-9		
49	Cytochalasin B	F actin capper	5mg/mL	14930-96-2		
50	EHNA HCl	Phosphodiesterase (PDE2) inhibitor/adenosine deaminase inhibitor	5mg/mL	51350-19-7		
50	Go6976	PKC inhibitor	5mg/mL		n=7/8 abnormal	Go6976 (1:200)
50	KN-62	CaM kinase II inhibitor	5mg/mL	127191-97-3	n=5/8 misguided and n=2/8 reduced regrowth	
50	L-NAME	NO synthesis inhibitor	5mg/mL	51298-62-5		
50	Methotrexate	DHFR inhibitor	5mg/mL	59-05-2		
51	6,7-ADTN HBr	Dopamine agonist	5mg/mL	73304-33-3		
51	Pregnenolone 16α carbonitrile	PXR/SXR agonist	5mg/mL	1434-54-4	n=4/4 normal	
51	Estradiol	estrogen	5mg/mL	50-28-2		
51	H7	kinase inhibitor	5mg/mL	84477-87-2		

51	KT-5720	PKA inhibitor	0.5mg/mL	108068-98-0		
52	Decoyinine	lowers GTP levels	5mg/mL	2004-04-8		
52	ICRF-193	topo II inhibitor that does not cause DNA breaks	5mg/mL	21416-68-2	n=4/4 normal	
52	L-744,832	Ras farnesyltransferase inhibitor	5mg/mL			
52	5-Iodotubercidin	ERK-2 inhibitor	5mg/mL	24386-93-4		
52	Mitomycin C	cross links DNA	5mg/mL	50-07-7		
53	Deoxymannojirimycin(1)	mannosidase inhibitor	5mg/mL	84444-90-6		
53	Doxorubicin	topoisomerase II inhibitor, induces apoptosis	5mg/mL	25316-40-9	n=3/3 normal	
53	H9	kinase inhibitor	5mg/mL	116700-36-8		
53	HA1077	inhibitor of Rho-dependent kinases	5mg/mL	103745-39-7		
53	Indomethacin	cyclooxygenase inhibitor	5mg/mL	53-86-1		
54	Deoxynorjrimycin(1)	glucosidase inhibitor	5mg/mL	19130-96-2		
54	DRB	CKII inhibitor	5mg/mL	53-85-0	n=4/8 abnormal	
54	HA-1004	kinase inhibitor	5mg/mL	91742-10-8	n=2/8 misguided and n=2/8 reduced regrowth	HA-1004 (1:200)
54	IBMX	PDE inhibitor (broad spec), adenosineR agonist	5mg/mL	28800-58-4		
54	Lavendustin A	Tyrosine kinase inhibitor EGF-R)	5mg/mL	125697-92-9		Lavendustin A (1:200)
55	Dexamethasone	corticosteroid	5mg/mL	50-02-2		Dexamethasone (1:200)
55	MnTBAP	SOD mimetic	5mg/mL		n=4/8 abnormal	MnTBAP (1:200)
55	Genistein	Tyrosine kinase inhibitor	5mg/mL	446-72-0	n=3/8 misguided and n=1/8 reduced regrowth	
55	N9-Isopropylolomoucine	CDC-2 kinase inhibitor	5mg/mL			
55	Monastrol	Eg5 inhibitor	5mg/mL			
56	E6 Berbamine	calmodulin inhibitor	5mg/mL			
56	HBDDE	PKC inhibitor	5mg/mL		n=4/4 normal	
56	Leupeptin	protease inhibitor	5mg/mL	103476-89-7		
56	E-64-d	calpain/cathepsin inhibitor	5mg/mL	88321-09-9		
56	GM6001	broad spectrum MMP inhibitor	5mg/mL			
57	Dibutyrylcyclic AMP	PKA activator	5mg/mL	16980-89-5		
57	MCI-186	antioxidant, cytoprotectant	5mg/mL	89-25-8	n=4/4 normal	
57	Dibutyrylcyclic GMP	PKA activator	5mg/mL	51116-00-8		
57	Histamine	Histamine receptor agonist	5mg/mL	51-74-1		
57	RWJ-60475-(AM)3	CD45 phosphatase inhibitor	5mg/mL			
58	HNMPA-(AM)3	Insulin receptor TK inhibitor	5mg/mL			

58	LFM-A13	BTK inhibitor	5mg/mL		tested fish died	
58	FK-506	FKBP ligand	5mg/mL	104987-11-3	pool toxic	
58	Phenanthridinone [6(5H)-Phenanthridinone]	PARP inhibitor	5mg/mL	1015-89-0		
58	Puromycin	protein synthesis inhibitor	5mg/mL	3506-23-8		
59	OBAA	phospholipase A2 inhibitor	5mg/mL			
59	SB-431542	ALK4, ALK5, ALK7 inhibitor	5mg/mL	301836-41-9	n=7/8 normal	
59	SQ22536	adenylate cyclase inhibitor	5mg/mL	17318-31-9		
59	Tyrphostin AG-825	HER-1,2 tyrosine kinase inhibitor	5mg/mL			
59	Z-VAD-FMK	Caspase inhibitor (broad spectrum)	5mg/mL			
60	SB 203580	Suppressor of MAPKAP kinase-2	5mg/mL	152121-47-6		SB 203580 (1:100)
60	Alrestatin	aldose reductase inhibitor	5mg/mL	51411-04-2	n=4/4 abnormal	
60	Kavain (±)	voltage-dependent Na channel inhibitor	5mg/mL	500-64-1	n=2/4 misguided and n=2/4 reduced regrowth	
60	U-0126	MEK inhibitor	5mg/mL			U-0126 (1:300)
60	PD 98059	MEK inhibitor	5mg/mL	167869-21-8		
61	Pifithrin	p53 inhibitor	5mg/mL	63208-82-2		Pifithrin (1:100)
61	CITCO	Const. androstane receptor (CAR) agonist	5mg/mL		n=4/4 abnormal	
61	SP-600125	JNK inhibitor	5mg/mL	129-56-6	n=4/4 reduced regrowth	SP-600125 (n=4/4 reduced regrowth, 1:100)
61	Quercetin	kinase inhibitor (plus other)	5mg/mL	6151-25-3		
61	SB 202190	MAP kinase inhibitor	5mg/mL			SB 202190 (1:100)
62	Olomoucine	CDK inhibitor	5mg/mL	101622-51-9		
62	Piroxicam	COX1 inhibitor	5mg/mL	36322-90-4	n=4/4 normal	
62	Swainsonine	protein glycosylation inhibitor	5mg/mL	72741-87-8		
62	Vinblastine	tubulin inhibitor	5mg/mL	143-67-9		
62	Mycophenolic acid	Inosine-5'-monophosphate dehydrogenase inhibitor	5mg/mL	24280-93-1		
63	Ouabain	Na+K+ATPase inhibitor	5mg/mL	11018-89-6		
63	Cyclopamine	Hedgehog pathway inhibitor	5mg/mL	4449-51-8	n=4/4 normal	
63	Cyclo [Arg-Gly-Asp-D-Phe-Val]	integrin inhibitor	5mg/mL			
63	Serotonin	serotonin receptor agonist	5mg/mL	153-98-0		
63	W7	calmodulin antagonist	5mg/mL	61714-27-0		
64	Diazoxide	Potassium channels	5mg/mL	364-98-7		
64	Minoxidil sulfate	Potassium channels	5mg/mL	83701-22-8	n=4/4 normal	
64	RHC-80267	DAG lipase inhibitor	5mg/mL	83654-05-1		

64	Resveratrol	SIRT1 activator	5mg/mL	501-36-0		
64	Y-27632	ROCK inhibitor	5mg/mL	146986-50-7		
64	Xestospongin C	IP3 receptor blocker	0.5mg/mL			
65	Prazocin	adrenoreceptor agonist	5mg/mL	19216-56-9		
65	Thalidomide	TNFalpha synthesis inhibitor	5mg/mL	50-35-1	n=4/4 normal	
65	Tyrphostin AG-126	tyrosine kinase inhibitor	5mg/mL			
65	Yohimbine	Adrenoreceptor antagonist (alpha)	5mg/mL	146-48-5		
65	Cerulenin	Fatty acid biosynthesis inhibitor	5mg/mL	17397-89-6		
66	Propidium iodide	DNA intercalator	5mg/mL	25535-16-4		
66	Roscovitine	CDK inhibitor	5mg/mL		n=4/4 abnormal	Roscovitine (1:500)
66	Thiocitrulline [L-Thiocitrulline HCl]	bNOS inhibitor	5mg/mL	156719-37-8	n=1/4 misguided and n=3/4 reduced regrowth	
66	Tyrphostin 1	Calcineurin inhibitor	5mg/mL	2826-26-8		Tyrphostin 1 (n=4/4 reduced regrowth, 1:1000)
66	Z-Leu3-VS	Proteasome inhibitor	5mg/mL			
67	Pepstatin	protease inhibitor	5mg/mL	26305-03-3		
67	Propranolol (S-)	adrenoceptor antagonist (beta)	5mg/mL	4199-10-4	n=4/4 normal	
67	Piceatannol	Syk inhibitor	5mg/mL	10083-24-6		
67	GF-109203X	PKC inhibitor	5mg/mL	133052-90-1		
67	ZM336372	Raf inhibitor	5mg/mL			
68	6-Formylindolo [3,2-B] carbazole	AHR agonist	1mM	172922-91-7		
68	NS-1619	Potassium channels	5mg/mL	153587-01-0	n=3/4 abnormal	
68	Phentolamine	Potassium channels	5mg/mL	73-05-2	n=3/4 reduced regrowth	
68	10-Hydroxycamptothecin	topoisomerase 1 inhibitor	5mg/mL	64439-81-2		10-Hydroxycamptothecin (n=4/4 reduced regrowth, 1:600)
68	Ro 31-8220	PKC inhibitor	5mg/mL	138489-18-6		
69	Juglone	PIN1 inhibitor	5mg/mL	481-39-0		
69	Rapamycin	FRAP inhibitor	5mg/mL	53123-88-9	n=2/4 abnormal	none of the individual compounds caused abnormal regrowth
69	Indirubin	GSK-3beta inhibitor	5mg/mL	479-41-4	n=2/4 reduced regrowth	
69	Nafamostst mesylate	Serine protease inhibitor	5mg/mL	82956-11-4		

toxic compounds excluded from further testing

1,25-Dihydroxyvitamin D3	Vitamin D receptor agonist	1mM	32222-06-3
24,25-Dihydroxyvitamin D3	Vitamin D receptor ligand	1mM	40013-87-4
25-Hydroxyvitamin D3	Vitamin D receptor ligand	1mM	19356-17-3
C8 Ceramide	Stimulates Cer-activated PK	1mM	
REV-5901	5-Lipoxygenase inhibitor	1mM	101910-24-1
TTNPB	Retinoid RAR agonist	1mM	71441-28-6
Aconitine	Sodium channels	5mg/mL	302-27-2
Amiodarone	Calcium channels	5mg/mL	1951-25-3
A-23187	Calcium ionophore	5mg/mL	52665-69-7
Bay K-8644	Calcium channels	5mg/mL	98791-67-4
Bepridil	Calcium channels	5mg/mL	74764-40-2
Cyclopiazonic acid	Intracellular calcium	5mg/mL	18172-33-3
Fipronil	Misc. channels	5mg/mL	120068-37-3
Flufenamic acid	Potassium channels	5mg/mL	530-78-9
Flunarizine	Calcium channels	5mg/mL	30484-77-6
Fluspiriline	Potassium channels	5mg/mL	1841-19-6
FPL-64176	Calcium channels	5mg/mL	120934-96-5
Nicardipine	Calcium channels	5mg/mL	54527-84-3
Niflumic acid	Misc. channels	5mg/mL	4394-00-7
Niguldipine	Calcium channels	5mg/mL	113317-61-6
Nimodipine	Calcium channels	5mg/mL	66085-59-4
Nitrendipine	Calcium channels	5mg/mL	39562-70-4
N-Phenylanthranilic acid	Misc. channels	5mg/mL	91-40-7
NPPB	Misc. channels	5mg/mL	107254-86-4
Paxilline	Potassium channels	5mg/mL	57186-25-1
Penitrem A	Potassium channels	5mg/mL	12627-35-9
Pimozide	Calcium channels	5mg/mL	2062-78-4
Ryanodine	Intracellular calcium	5mg/mL	15662-33-6
SDZ-201106	Sodium channels	5mg/mL	97730-95-5
SKF-96365	Calcium channels	5mg/mL	130495-35-1
Thapsigargin	Intracellular calcium	5mg/mL	67526-95-8

Veratridine	Sodium channels	5mg/mL	71-62-5
ZM226600	Potassium channels	5mg/mL	
Tosyl-Phe-CMK (TPCK)	Serine protease inhibitor	5mg/mL	402-71-1
3,4-Dichloroisocoumarin	Granzyme B inhibitor	5mg/mL	51050-59-0
HA14-1	Bcl-2 ligand induces apoptosis	5mg/mL	
Decylubiquinone	inhibits mitochondrial permeability pore opening	5mg/mL	55486-00-5
Triptolide	inhibits NFkappaB transcriptional activation	5mg/mL	38748-32-2
Nigericin	induces intracellular acidification	5mg/mL	28380-24-7
β-Lapachone	topoisomerase 1 inhibitor	5mg/mL	4707-32-8
Parthenolide	IkappaB kinase inhibitor	5mg/mL	20554-84-1
Helenalin	NFkappaB inhibitor	5mg/mL	6754-13-8
U-74389G	superoxide/free-radical inhibitor	5mg/mL	153190-29-5
Ebselen	glutathione peroxidase mimetic	5mg/mL	60940-34-3
Zardaverine	phosphodiesterase (PDE1/2) inhibitor	5mg/mL	101975-10-4
Vinpocetine	phosphodiesterase (PDE1) inhibitor	5mg/mL	42971-09-5
Trequinsin	phosphodiesterase (PDE3) inhibitor	5mg/mL	78416-81-6
Rolipram	phosphodiesterase (PDE4) inhibitor	5mg/mL	61413-54-5
MY-5445	phosphodiesterase (PDE5) inhibitor	5mg/mL	78351-75-4
AG1478	Tyrosine kinase inhibitor. Broad spectrum	5mg/mL	153436-53-4
AG-879	NGF receptor inhibitor	5mg/mL	
Ikarugamyin	inhibits clathrin coated pit mediated endocytosis	5mg/mL	36531-78-9
Amino-1,8-naphthalimide [4-Amino-1,8-naphthalimide]	PARP inhibitor	5mg/mL	1742-95-6
17-Allylamino-geldanamycin	HSP-90 inhibitor	5mg/mL	75747-14-7
Aristolochic acid	phospholipase A2 inhibitor	5mg/mL	313-67-7
MBCQ	phosphodiesterase (PDE5) inhibitor	5mg/mL	
Bafilomycin A1	vaculolar ATPase inhibitor	0.5mg/mL	88899-55-2
Blebbistatin	Myosin II inhibitor	5mg/mL	
Brefeldin A	ARF GEF inhibitor	5mg/mL	20350-15-6
Bromo-7-nitroindazole [3-Bromo-7-nitroindazole]	NO synthase inhibitor	5mg/mL	74209-34-0
Calyculin A	PP1, PP2A inhibitor	0.5mg/mL	101932-71-2
Camptothecin	Topoisomerase 1 inhibitor	5mg/mL	7689-03-4
Cantharidin	PP2A inhibitor	5mg/mL	56-25-7
Dipyridamole	cGMP phosphodiesterase inhibitor	5mg/mL	58-32-2

CGP-37157	inhibitor of mitochondrial Na+Ca+2 exchange	5mg/mL	75450-34-9
CAPE	Antioxidant/ NFkappa B inhibitor	5mg/mL	104594-70-9
Clozapine	Dopamine antagonist	5mg/mL	5786-21-0
Curcumin	NFkappaB inhibitor	5mg/mL	458-37-7
Cycloheximide	protein synthesis inhibitor	5mg/mL	66-81-9
Cypermethrin	calcineurin inhibitor	5mg/mL	52315-07-8
Cytochalasin D	F actin capper	5mg/mL	22144-77-0
D609	PC-PLC inhibitor	5mg/mL	83373-60-8
Damnacanthal	p56lck inhibitor	5mg/mL	477-84-9
YC-1	GC stimulator / Hif-1 alpha inhibitor	5mg/mL	
GW-9662	PPAR gamma antagonist	5mg/mL	22978-25-2
Hinokitiol	Iron chelator	5mg/mL	499-44-5
Diphenyleneiodonium Cl	flavoprotein inhibitor	5mg/mL	10182-84-0
2,5-Ditertbutylhydroquinone	ER Ca++ ATPase inhibitor	5mg/mL	88-58-4
Etoposide	topoisomerase II inhibitor	5mg/mL	33419-42-0
FCCP	mitochondrial uncoupler	5mg/mL	370-86-5
Forskolin	Adenylate cyclase activator	5mg/mL	66575-29-9
Geldanamycin	HSP90 inhibitor	5mg/mL	30562-34-6
Nimesulide	Cox 2 inhibitor	5mg/mL	51803-78-2
H-89	PKA inhibitor	5mg/mL	127243-85-0
Hoechst 33342 (cell permeable) (BisBenzimide)	DNA minor groove binder	5mg/mL	23491-52-3
Huperzine A [(-)-Huperzine A]	acetylcholinesterase inhibitor	5mg/mL	102518-79-6
24(S)-Hydroxycholesterol	LXR agonist	5mg/mL	
Ionomycin	Ca++ ionophore	5mg/mL	56092-81-0
NSC-95397	CDC25 phosphatase inhibitor	5mg/mL	93718-83-3
K252A	Kinase inhibitor (Broad spectrum)	0.5mg/mL	97161-97-2
Furoxan	NO donor	5mg/mL	
Latrunculin B	Actin inhibitor	5mg/mL	76343-94-7
Bay 11-7082	Inhibits IKK kinase activation	5mg/mL	195462-67-7
Tyrphostin-8	Calcineurin inhibitor	5mg/mL	
LY-294002	PI-3-Kinase inhibitor	5mg/mL	154447-36-6
LY-83583	Inhibits NO-activation of guanylate cyclase	5mg/mL	91300-60-6
Manoalide	Phospholipase A2 inhibitor	5mg/mL	75088-80-1

Manumycin A	ras farnesylation inhibitor	5mg/mL	52665-74-4
Mastoparan	activates heterotrimeriuc GTPases	5mg/mL	72093-21-1
Gliotoxin	Inhibitor of 20S-proteasome chymotrypsin activity	5mg/mL	67-99-2
Epibatidine (±)	nicotinic cholinergic agonist	5mg/mL	140111-52-0
Mevinolin (Lovastatin)	Inhibitor HMG-CoA reductase	5mg/mL	75330-75-5
ML7	kinase inhibitor	5mg/mL	109376-83-2
ML9	kinase inhibitor	5mg/mL	105637-50-1
GW-5074	cRAF1 kinase inhibitor	5mg/mL	220904-83-6
Monensin	Na+ ionophore	5mg/mL	22373-78-0
Nocodazole	tubulin inhibitor	5mg/mL	31430-18-9
Oligomycin A	Fo ATP synthase inhibitor	5mg/mL	579-13-5
PCA 4248	PAF antagonist	5mg/mL	
SU-4312	VEGF-R (Flk-1) tyrosine kinase	5mg/mL	5812-07-7
Phorbol 12 myristate 13 acetate	PKC activator	5mg/mL	16561-29-8
PP1	src family trosine kinase inhibitor	5mg/mL	
RK-682	VHR phosphatase inhibitor	5mg/mL	
Rottlerin	PKC delta inhibitor	5mg/mL	82-08-6
Indirubin-3'-monoxime	GSK-3beta inhibitor	5mg/mL	
Splitomycin	sir2p inhibitor	5mg/mL	
PP2	Src family tyrosine kinase inhibitor	5mg/mL	
Staurosporine	kinase inhibitor - non-selective	5mg/mL	62996-74-1
Tamoxifen	estrogen antagonist	5mg/mL	54965-24-1
RG-14620	EGF-R tyrosine kinase inhibitor	5mg/mL	136831-49-7
TPEN	cell permable heavy metal chelator	5mg/mL	16858-02-9
Trichostatin-A	histone deacetylase inhibitor	5mg/mL	58880-19-6
Trifluoperazine	calmodulin inhibitor-possibly only at high concentrations!	5mg/mL	440-17-5
Tunicamycin	glycosylation inhibitor	5mg/mL	11089-65-9
Tyrphostin 9	PDGF-R tyrosine kinase inhibitor	5mg/mL	10537-47-0
U73122	PLC inhibitor	5mg/mL	112648-68-7
Valinomycin	K+ ionophore	5mg/mL	2001-95-8
Wortmannin	PI-3Kinase, other kinases inhibitor	5mg/mL	19545-26-7
NS-398	Cox-2 inhibitor	5mg/mL	123653-11-2
AA-861	5-lipoxygenase inhibitor	5mg/mL	80809-81-0

Shikonin	Apoptosis inducer, p53 dependent	5mg/mL	517-89-5
CDC	12-Lipoxygenase inhibitor	5mg/mL	132465-11-3
Phenoxybenzamine	Calmodulin antagonist	5mg/mL	63-92-3
Wiskostatin	N-WASP inhibitor	5mg/mL	
